# Traumatic brain injury in mice induces changes in the expression of the XCL1/XCR1 and XCL1/ITGA9 axes

**DOI:** 10.1007/s43440-020-00187-y

**Published:** 2020-11-13

**Authors:** Agata Ciechanowska, Katarzyna Popiolek-Barczyk, Katarzyna Ciapała, Katarzyna Pawlik, Marco Oggioni, Domenico Mercurio, Maria-Grazia de Simoni, Joanna Mika

**Affiliations:** 1 Department of Pain Pharmacology, Maj Institute of Pharmacology, Polish Academy of Sciences, 12 Smetna Str., 31-343 Kraków, Poland; 2 Department of Neuroscience, Istituto di Ricerche Farmacologiche Mario Negri IRCCS, via Mario Negri, 2, 20156 Milan, Italy

**Keywords:** TBI, Chemokine, XCL1, XCR1, ITGA9, Microglia, Astroglia

## Abstract

**Background:**

Every year, millions of people suffer from various forms of traumatic brain injury (TBI), and new approaches with therapeutic potential are required. Although chemokines are known to be involved in brain injury, the importance of X-C motif chemokine ligand 1 (XCL1) and its receptors, X-C motif chemokine receptor 1 (XCR1) and alpha-9 integrin (ITGA9), in the progression of TBI remain unknown.

**Methods:**

Using RT-qPCR/Western blot/ELISA techniques, changes in the mRNA/protein levels of XCL1 and its two receptors, in brain areas at different time points were measured in a mouse model of TBI. Moreover, their cellular origin and possible changes in expression were evaluated in primary glial cell cultures.

**Results:**

Studies revealed the spatiotemporal upregulation of the mRNA expression of *XCL1, XCR1* and *ITGA9* in all the examined brain areas (cortex, thalamus, and hippocampus) and at most of the evaluated stages after brain injury (24 h; 4, 7 days; 2, 5 weeks), except for *ITGA9* in the thalamus. Moreover, changes in XCL1 protein levels occurred in all the studied brain structures; the strongest upregulation was observed 24 h after trauma. Our in vitro experiments proved that primary murine microglial and astroglial cells expressed XCR1 and ITGA9, however they seemed not to be a main source of XCL1.

**Conclusions:**

These findings indicate that the XCL1/XCR1 and XCL1/ITGA9 axes may participate in the development of TBI. The XCL1 can be considered as one of the triggers of secondary injury, therefore XCR1 and ITGA9 may be important targets for pharmacological intervention after traumatic brain injury.

**Graphic abstract:**

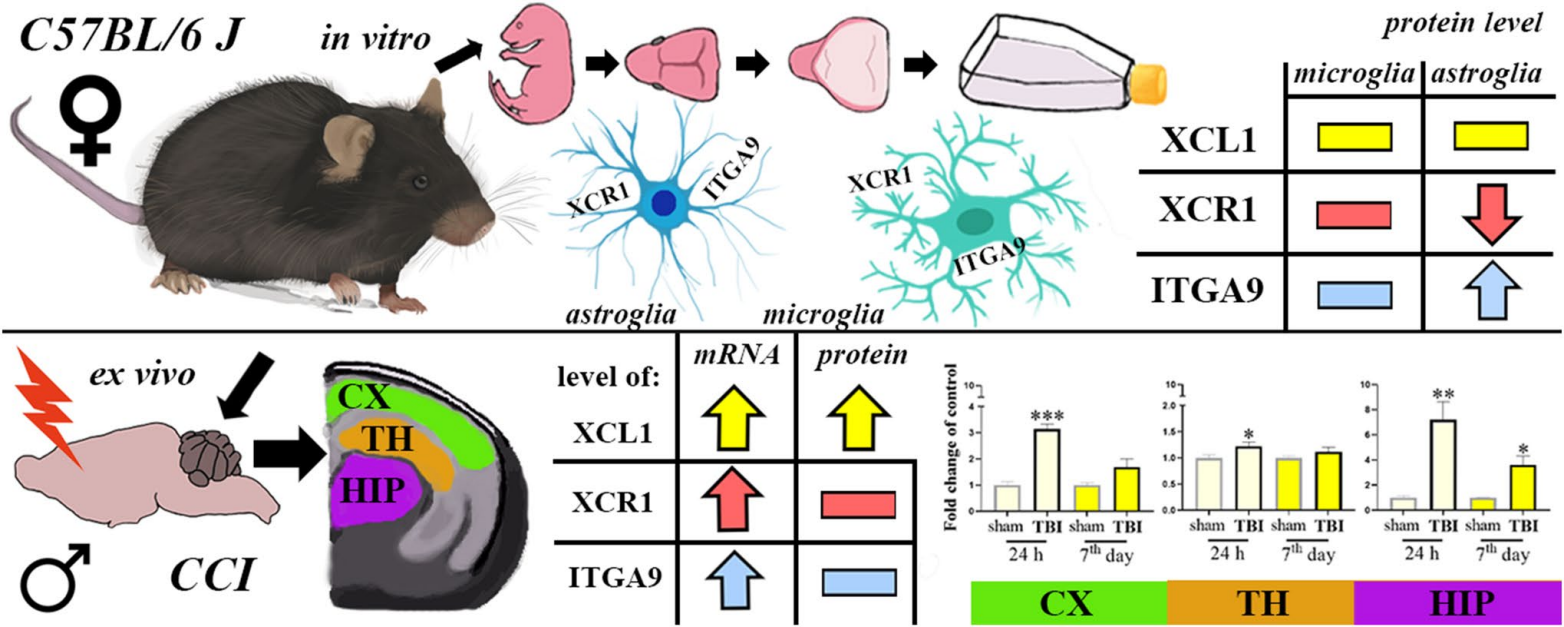

## Introduction

Traumatic brain injury (TBI) is a condition that is caused by sudden damage to the central nervous system (CNS) due to accidents, violence, or sport activity and is a major urgent medical need. TBI is extremely difficult to treat, since it leads to secondary injury as a consequence of blood–brain barrier (BBB) disruption, cell death, ischemia, and hemorrhage [1]. Because the commonly used therapies are insufficient and complicated, new approaches that are based on the identification of new, potential therapeutic targets could help us develop more accurate reactions in an effort to address the consequences of TBI. Neuronal damage in brain structures leads to primary cell death, which is induced directly by the trauma, and to the subsequent death of neurons caused by secondary cascades [2]. The complexity of the neuroimmunological responses that appear after TBI still needs to be understood. It is well established that cytokines play a key role in homeostasis [3]. It is known that many interleukins (e.g., IL-1β, IL-6, IL-18) are sharply upregulated in TBI patients [4–6]. Similar results were obtained in rodent models of TBI, and recent experimental studies have shown significant changes in some chemokines, e.g., CCL2, CCL3, CCL4, CCL9, CCL11, CX3CL1, and CXCL5 [1, 7, 8], however, there is still a lack of knowledge about X-C motif chemokine ligand 1 (XCL1, also known as lymphotactin and SCM-1alpha). XCL1 acts through X-C motif chemokine receptor 1 (XCR1), which is a G-protein coupled receptor [9] that has been detected in neurons [10, 11]. It was shown that after mental nerve damage the XCR1 is upregulated at the site of the injury—the authors proposed XCL1 as an excitability factor in orofacial pain [11]. Importantly, XCL1 is produced not only by immune cells [12], but also by neurons [10, 11, 13, 14]. Therefore, the role of XCL1 in TBI is particularly interesting. However, XCR1 is not the only receptor for XCL1. Interestingly, in 2017 Matsumoto et al. showed that XCL1 affects fibroblast migration through alpha-9 integrin (ITGA9) [15]. This receptor has been already detected in some cell types, including endothelial cells, epithelial cells, muscle cells, neutrophils and neural precursor cells [16–19]. However, the expression of ITGA9 on microglial and astroglial cells has not been studied thus far. In vitro studies showed that the neuronal expression of ITGA9 enhanced the ability of axons to regenerate [20]. On the other hand, it was suggested that blocking ITGA9 can be used as a therapeutic strategy in autoimmune diseases [21]. However, in the current literature, there is still a lack of knowledge about the roles of the XCL1/XCR1 and XCL1/ITGA9 axes in TBI.

Therefore, the aim of our study was to examine, using RT-qPCR, Western blot and ELISA techniques, the possible temporal changes in the mRNA and protein levels of XCL1 and its two receptors, XCR1 and ITGA9, in different brain structures (cortex, thalamus, and hippocampus) after TBI. The injury was induced in mice by controlled cortical impact (CCI), which is a clinically relevant model of human TBI [22]. This model induces local responses in the brain tissue, leading to neuronal loss, BBB disruption and subsequent inflammatory response induction, including chemokine release [2]. Moreover, the goal of the study was to identify the cellular origin of XCL1 and to demonstrate the expression of XCR1 and ITGA9 in LPS-stimulated primary cultures of microglia and astrocytes using RT-qPCR, Western blot and ELISA techniques. Additionally, the influence of XCL1 on primary cell cultures of microglia and astrocytes was tested.

## Materials and methods

### Animals

In this study, the C57BL/6J mice from the Charles River, Italy, Germany were used, as follows (1) adult males for the TBI model (9–11 weeks old, weighing 22–27 g); (2) 1-day-old mice pups for primary glial cell cultures studies. The mice were housed at a temperature of 22 ± 2 °C and a relative humidity 55 ± 10% with a 12-h light/dark cycle. The mice were housed 4–5 per cage and were given ad libitum access to food and water. All the procedures using animals were performed in agreement with institutional guidelines and in compliance with national and international laws and policies.

### Induction of the traumatic brain injury model

The animals were anesthetized with inhalation anesthesia (isoflurane—induction, 3%; maintenance, 1.5%) in N_2_O/O_2_ (70/30%) and immobilized in a stereotaxic frame. Next, the mice were subjected to craniotomy on the left side and then to TBI by CCI, according to a previously described procedure [23]. This model reliably causes TBI, which was confirmed by many studies performed in The IRCCS-Istituto di Ricerche Farmacologiche Mario Negri [23, 24]. This model of severe TBI is typically associated with minimal/no mortality [23]. The assessment of sensorimotor deficits was confirmed as a rule after CCI with the use of composite neurosurgery and the beam walk test weekly for 4 weeks [23]. Contusion volume after these TBI model was observed and calculated in perfused, frozen and cryosected brains stained with cresyl violet as previously described [24]. The TBI model used a pneumatic piston stably mounted at an angle of 20° from the vertical plane. This piston drove a rigid 3-mm impactor that applied force perpendicularly to the exposed dura mater over the left parietotemporal cortex at a velocity of 5 m/s and a depth of 1 mm. Cranioplasty was performed after craniotomy, and the scalp was sutured. The sham-injured mice were subjected to identical anesthesia and surgery but were not subjected to brain injury. There was no loss in the number of animals after surgery.

### Primary microglial and astroglial cell cultures

The in vitro studies were performed using primary microglial and astroglial cell cultures prepared from the cerebral cortex obtained from newborn C57BL/6J mice, as described in our previous paper [8]. The cells were seeded at a density of 3 × 10^5^ cells/cm^2^ in culture medium consisting of high-glucose GlutaMAX™ DMEM supplemented with 10% heat-inactivated fetal bovine serum, 0.1 mg/ml streptomycin and 100 U/ml penicillin (Gibco, New York, USA) in poly-l-lysine-coated 75-cm^2^ culture flasks. The cells were grown in a 37 °C incubator with a humidified atmosphere of 5% CO_2_ in air. The culture medium was replaced after 4 days. On day 16, the microglial cells, which were loosely attached to the monolayer, were harvested by gentle shaking (70 rpm for 1 h and 90 rpm for 15 min) and centrifugation (800 rpm for 10 min), and the cell viability was determined using the trypan blue (Bio-Rad, Warsaw, Poland) exclusion method. Then, fresh medium was added to the same culture bottles. After a few days, the astroglial cells were prepared by shaking the flasks for 4 h and trypsinization using a 0.05% trypsin–EDTA solution (Sigma-Aldrich, Saint Louis, USA). For the protein analysis, both microglia and astroglia were seeded at a density of 1.2 × 10^6^ cells/well in 6-well plates and incubated for 48 h before further experiments. The IBA-1 and GFAP markers were used to assess cell purity. Only the minimal essential number of animals was used, and all of the procedures were performed according to the recommendations of the NIH Guide for the Care and Use of Laboratory Animals. The cells were treated with LPS (lipopolysaccharide from *Escherichia coli* 0111:B4; Sigma-Aldrich, St. Louis, USA), which is causing the oxidative stress and inflammatory status [25, 26]. The dose (100 ng/ml) of LPS and time point (24 h) was chosen based on our previous studies [8, 27]. Moreover, cells were treated with XCL1 (recombinant mouse XCL1 Protein; R&D Systems, Minneapolis, USA) (200 ng/ml) or vehicle (0.2% BSA in PBS). The dose was selected according to previously reported studies [28].

### Biochemical tests

#### Analysis of gene expression by RT-qPCR

For the RT-qPCR studies, selected brain areas were collected from the sham and TBI mice sacrificed at the following time points: 24 h, 4 days and 7 days, 2 weeks and 5 weeks. In addition, cell lysates from the primary microglial and astroglial cultures were collected and used for the study. Tissues from the ipsilateral cortex, hippocampus and thalamus were dissected, rapidly placed into RNAlater (Ambion, Inc., Austin, USA), frozen and stored at − 80 °C until use. For RT-qPCR, total RNA was extracted according to Chomczynski and Sacchi [29] with the TRIzol reagent (Invitrogen, Carlsbad, USA) as previously described [30]. The cell lysates were directly treated with the TRIzol reagent (Invitrogen, Carlsbad, USA). The RNA concentration was measured using the DeNovix DS-11 Spectrophotometer (DeNovix Inc., Wilmington, USA). Reverse transcription was performed with 1000 ng or 300 ng in case of primary glial cell cultures, of total RNA using Omniscript Reverse Transcriptase (Qiagen Inc., Hilden, Germany) at 37 °C for 60 min. The resulting cDNA was diluted 1:10 with H_2_O. RT-qPCR was performed using Assay-On-Demand TaqMan probes according to the manufacturer's protocol (Applied Biosystems, Foster City, USA) with an iCycler device (Bio-Rad, Hercules, Warsaw, Poland). The following TaqMan primers were used: Mm00446968_m1 (*Hprt*), Mm00434772_m1 (*Xcl1*), Mm00442206_s1 (*Xcr1*), Mm00519317_m1 (*Itga9*), Mm00434228_m1 (*IL1β*), Mm00446190_m1 (*IL-6*), Mm00434225_m1 (*IL-18*), Mm00441259_g1 (*CCL3*), Mm00443111_m1 (*CCL4*), Mm00441260_m1 (*CCL9*), Mm01288386_m1 (*IL-10*), and Mm01274147_g1 (*IL-18BP*). The expression of the *Hprt* transcript (a housekeeping gene) was quantified to control for variations in the amounts of cDNA. The cycle threshold values were automatically calculated using iCycler IQ 3.0 software with the default parameters. The abundance of RNA was calculated as 2^−(threshold cycle)^.

#### Enzyme-linked immunosorbent assay (ELISA) analysis

The cortical, thalamic and hippocampal tissues obtained at two time points (24 h and 7 days) after TBI or sham procedure and the cell culture lysates were used for enzyme-linked immunosorbent assays (ELISAs) according to the manufacturer’s recommendations. The tissue/cell lysates were fixed in RIPA buffer with a protease inhibitor cocktail (Sigma-Aldrich, St. Louis, USA). The level of XCL1 was measured in the tissue homogenates using the Mouse XCL1/Lymphotactin ELISA Kit (Sandwich ELISA, LS-F53223, LifeSpan Biosciences, Seattle, USA). The samples obtained from the cell cultures were measured by the Mouse XCL1/Lymphotactin ELISA Kit (Sandwich ELISA, LS-F39783, LifeSpan Biosciences, Seattle, USA). The detection ranges were as follows: LS-F53223: 6.25–400 pg/ml and LS-F39783: 62.5–4000 pg/ml. Positive controls for each assay were provided by the manufacturer.

#### Western blot analysis

The cortical, thalamic and hippocampal tissues obtained at two time points (24 h and 7 days) after TBI or sham procedure and the cell lysates from the primary microglial and astroglial cultures were collected and used for the study. The tissue/cell lysates were placed in RIPA buffer supplemented with a protease inhibitor cocktail (Sigma-Aldrich, St. Louis, USA). Then, the samples were cleared by 14,000×*g* centrifugation for 30 min at 4 °C. The total protein concentration was measured using the bicinchoninic acid (BCA) method. The protein samples (20 μg and 8 μg from the tissues and cells, respectively) were heated for 8 min at 98 °C in loading buffer (4 × Laemmli Buffer, Bio-Rad, Warsaw, Poland). Then, the samples were loaded in 4–15% Criterion TGX precast polyacrylamide gels (Bio-Rad, Warsaw, Poland) and, through the use of a semidry transfer system (30 min, 25 V), transferred to Immune-Blot PVDF membranes (Bio-Rad, Warsaw, Poland). Then, the membranes were blocked with dry milk (5%, nonfat, Bio-Rad, Warsaw, Poland) in Tris-buffered saline with 0.1% Tween 20 (TBST) for 1 h, washed with TBST (4 × 5 min), and incubated overnight at 4 °C with the following commercially available primary antibodies: mouse anti-GAPDH (1:5000; Merck, Darmstadt, Germany), rabbit anti-XCL1 (1:150; Novus Biologicals, Centennial, USA), rabbit anti-XCR1 (1:5000; Lifespan Biosciences, Seattle, USA), and rabbit anti-ITGA9 (1:3000; Abcam, Cambridge, Great Britain). Then, the membranes were incubated in horseradish peroxidase-conjugated anti-rabbit or anti-mouse secondary antibodies (Vector Laboratories, Burlingame, USA) at dilutions of 1:5000 for 1 h at room temperature. The primary and secondary antibodies were dissolved in solutions from the SignalBoost Immunoreaction Enhancer Kit (Merck, Darmstadt, Germany). The membranes were washed in TBST (4 × 5 min). The immune complexes were detected by the Clarity Western ECL Substrate (Bio-Rad, Warsaw, Poland) and visualized using the Fujifilm LAS-4000 Fluor Imager system. The quantification of the relative levels of the immunoreactive bands was performed using Fujifilm Multi Gauge.

### Statistical analysis

**Fig. 1:**
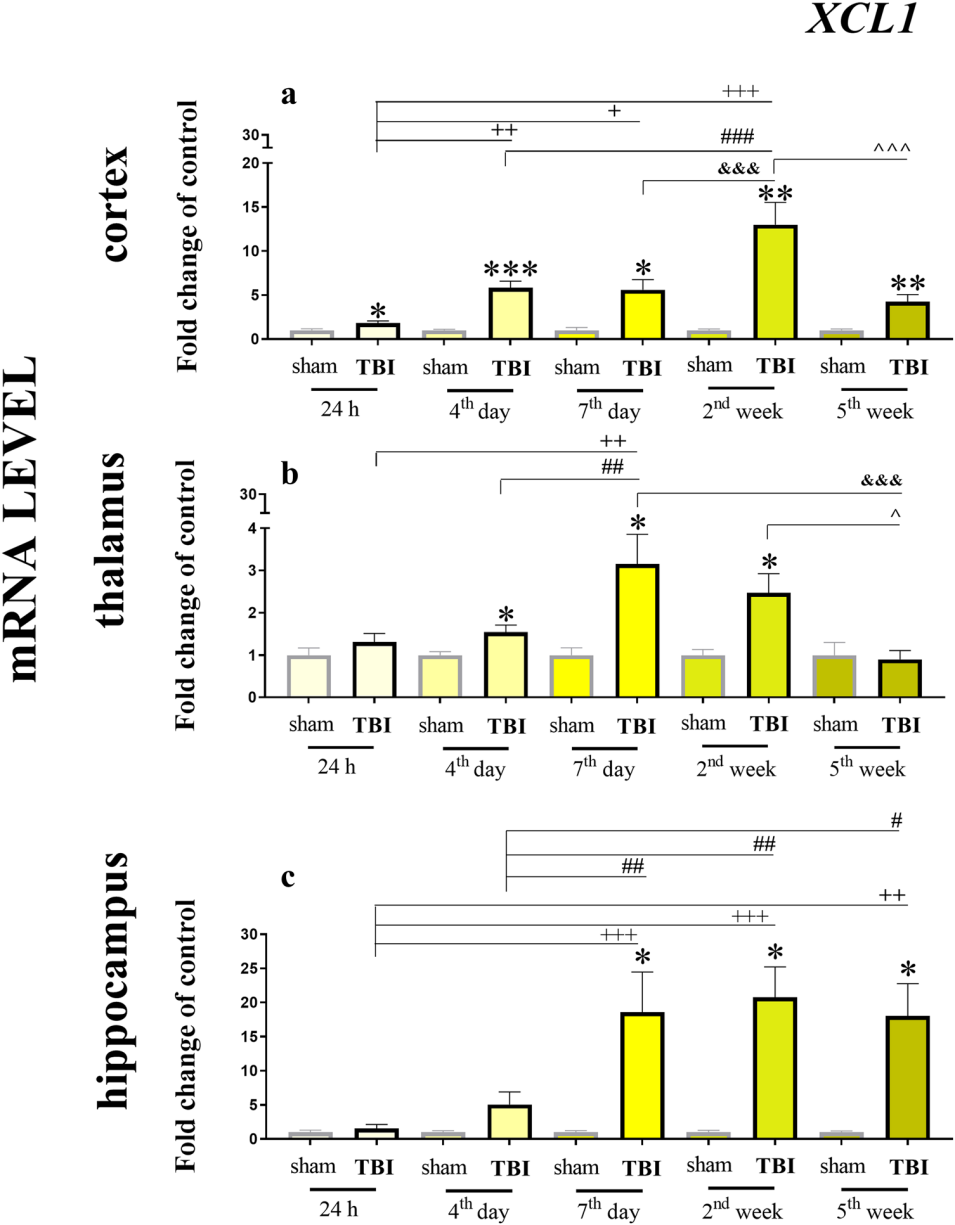
Time-dependent changes in *XCL1* mRNA expression in the cortex (**a**), thalamus (**b**) and hippocampus (**c**) of TBI or sham-injured mice at the selected time points. The data are presented as fold changes relative to the control (means ± SEMs.; sham groups *n* = 5–8; TBI groups *n* = 5–8). *p < 0.05; **p < 0.01; ***p < 0.001 indicate significant differences between the sham and TBI groups at each selected time point as evaluated by *t* test. ^+^*p* < 0.05; ^++^*p* < 0.01; ^+++^*p* < 0.001 shows significant differences comparing to the 24 h TBI group; ^#^*p* < 0.05; ^##^*p* < 0.01; ^###^*p* < 0.001 shows significant differences comparing to the 4th day TBI group; ^&&&^*p* < 0.001 shows significant differences comparing to the 7th day TBI group; ^*p* < 0.05; ^^^*p* < 0.001 shows significant differences comparing to the 2 weeks TBI group; as evaluated by two-way Anova

**Fig. 2:**
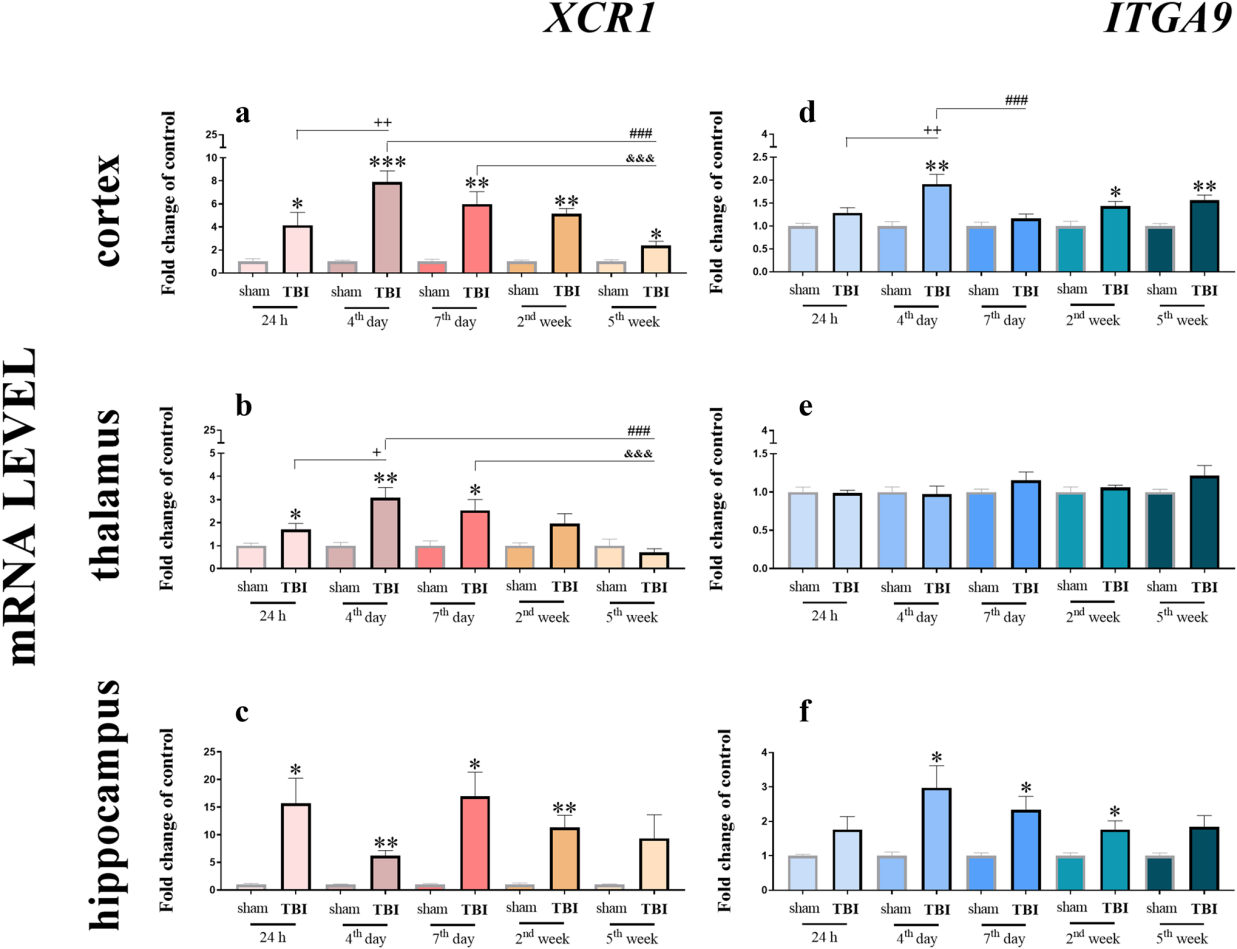
Time-dependent changes in *XCR1* and *ITGA9* mRNA expression in the cortex (**a**, **d**), thalamus (**b**, **e**) and hippocampus (**c**, **f**) of TBI or sham-injured mice at the selected time points. The data are presented as fold changes relative to the control (means ± SEMs; sham groups *n* = 5–8; TBI groups *n* = 5–8). *p < 0.05; **p < 0.01; ***p < 0.001 indicate significant differences between the sham and TBI groups at each selected time point as evaluated by *t* test. ^+^*p* < 0.05; ^++^*p* < 0.01 shows significant differences comparing to the 24 h TBI group; ^###^*p* < 0.001 shows significant differences comparing to the 4th day TBI group; ^&&&^*p* < 0.001 shows significant differences comparing to the 7th day TBI group; as evaluated by two-way Anova

**Fig. 3:**
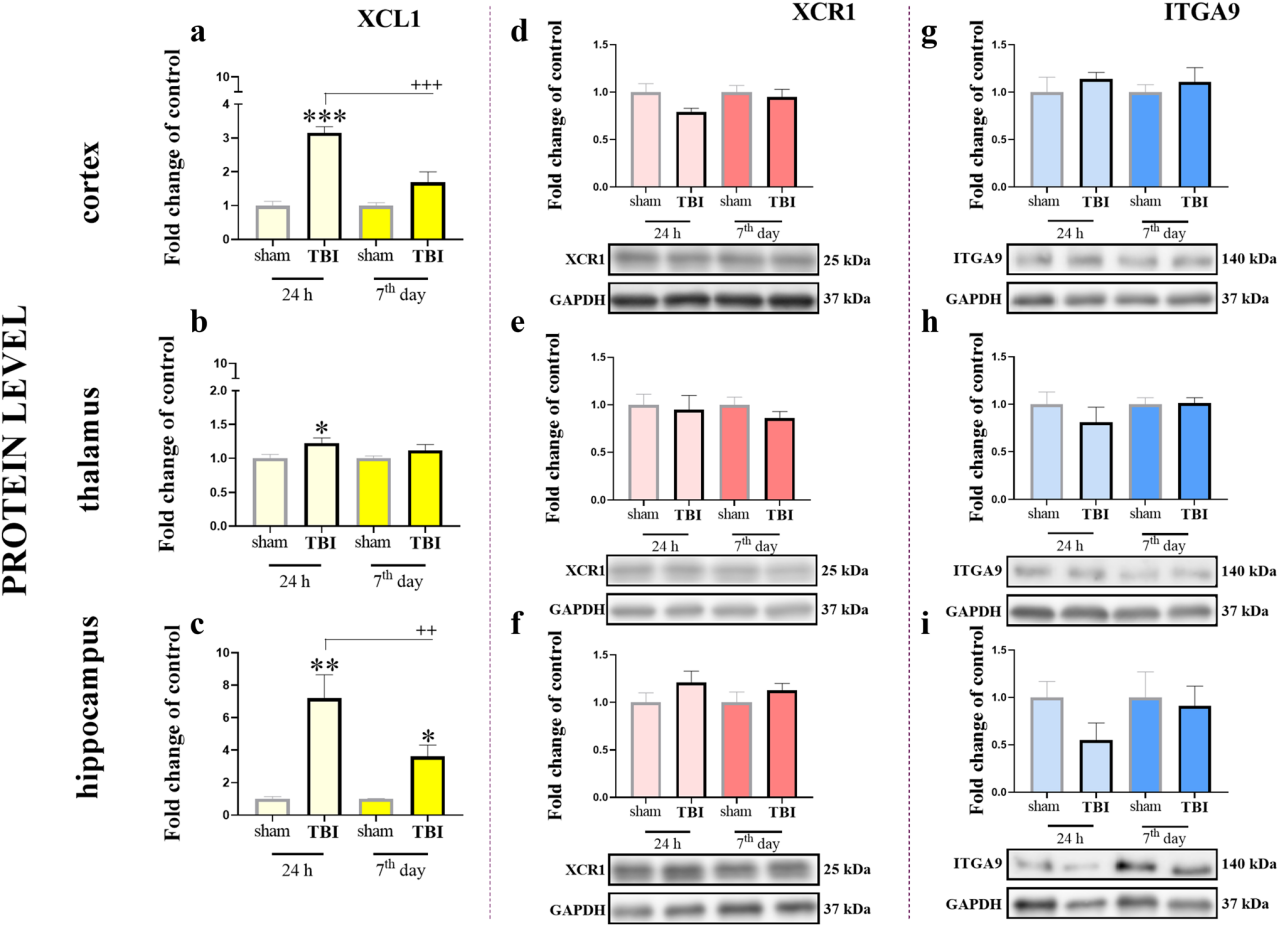
Time-dependent changes in the XCL1 (**a**–**c**), XCR1 (**d**–**f**) and ITGA9 (**g**–**i**) protein levels in the cortex (**a**, **d**, **g**), thalamus (**b**, **e**, **h**) and hippocampus (**c**, **f**, **i**) of TBI or sham-injured mice at the selected time points. The Elisa (**a**–**c**) and Western blot (**d**–**i**) data are presented as fold changes relative to the control (means ± SEMs; Elisa sham groups *n* = 6; TBI groups *n* = 5–6; Western blot sham groups *n* = 5–6; TBI groups *n* = 5–6). *p < 0.05; **p < 0.01; ***p < 0.001 indicate significant differences between the sham and TBI groups at each selected time point as evaluated by *t* test. ^++^*p* < 0.01; ^+++^*p* < 0.001 shows significant differences comparing to the 24 h TBI group; as evaluated by two-way Anova

**Fig. 4:**
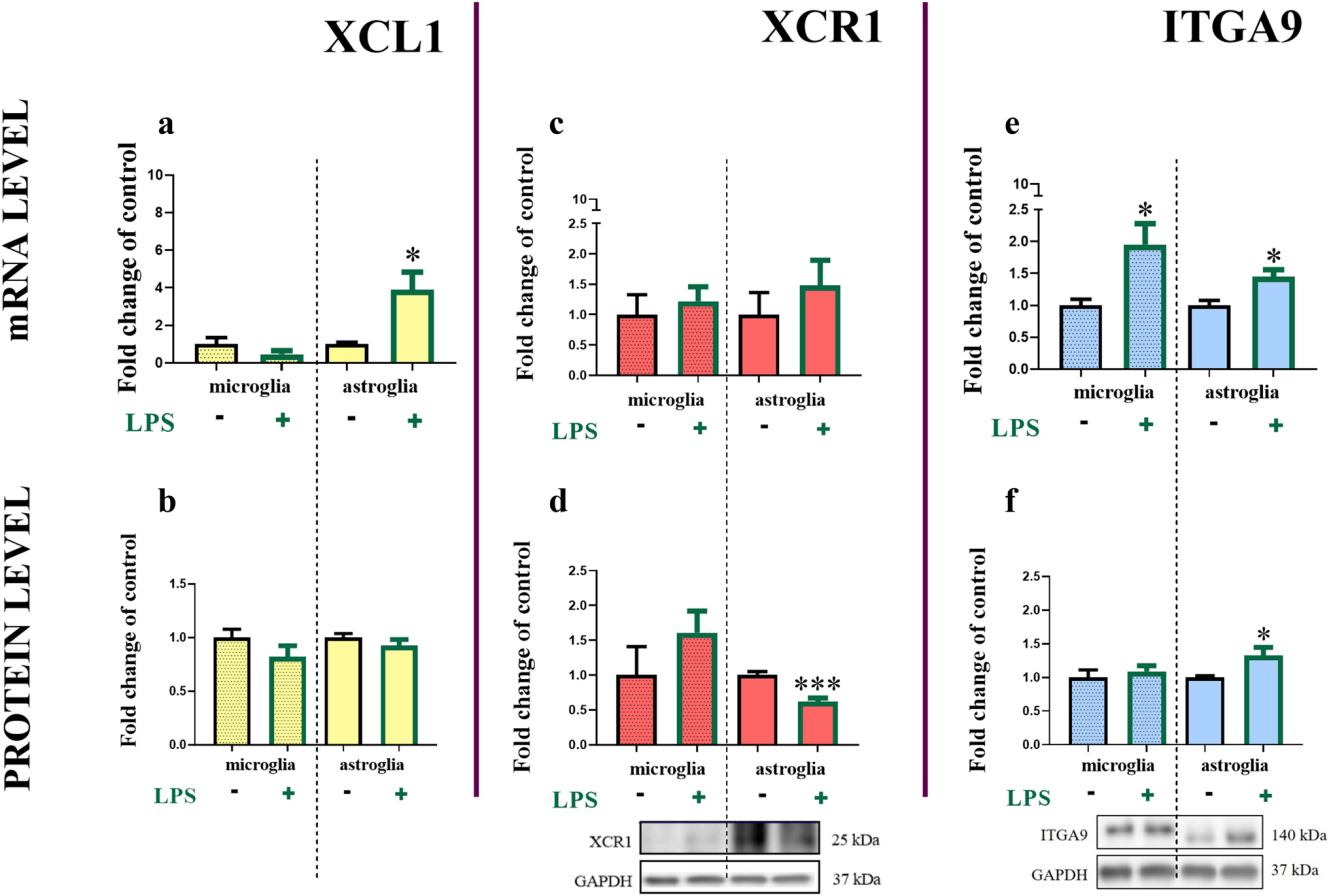
Changes in the mRNA and protein levels of XCL1 (**a**, **b**), XCR1 (**c**, **d**) and ITGA9 (**e**, **f**) in primary mouse microglial and astroglial cell cultures. The RT-qPCR (**a**, **c**, **e**)/Western blot (**d**, **f**)/ELISA (**b**) results are presented as fold changes relative to the control (means ± SEMs; RT-qPCR microglia group *n* = 3–7; astroglia group *n* = 3–7; Western blot microglia group *n* = 5–6; astroglia group *n* = 5–6; Elisa microglia group *n* = 6; astroglia group *n* = 5). *p < 0.05; ***p < 0.001 indicate significant differences between the unstimulated and LPS-stimulated microglial and astroglial cell cultures 24 h after treatment at the mRNA and protein levels as evaluated by *t* test

**Fig. 5:**
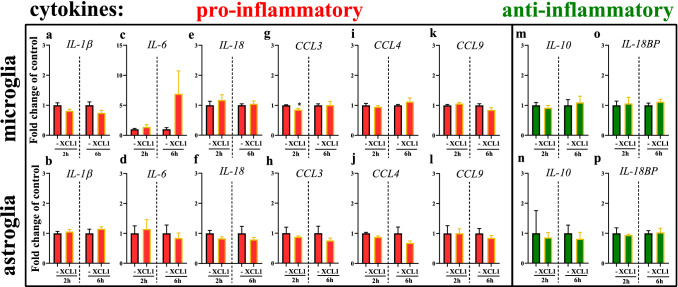
The effect of XCL1 on the mRNA levels (RT-qPCR) of pro- (*IL-1β*, *IL-6*, *IL-18*, *CCL3*, *CCL4*, *CCL9*) and anti- (*IL-10*, *IL-18BP*) inflammatory factors in microglial and astroglial cell cultures. The data are presented means ± SEM (*n* = 4 each group). *p < 0.05; indicate significant differences between the untreated and XCL1-treated (200 ng/ml) microglial and astroglial cell cultures, 2 and 6 h after treatment.at the mRNA level was evaluated by *t* test

The RT-qPCR results are presented as the normalized averages derived from the threshold cycle. For the tissue study (Figs. 1, 2, 3) and the primary cell culture study (Figs. 4, 5), the RT-qPCR/Western blot/ELISA results are presented as fold changes relative to the control [sham group (Figs. 1, 2, 3); unstimulated cells (Figs. 4, 5)]. All the results (mean ± SEM) were statistically evaluated using a *t* test (Figs. 1, 2, 3, 4, 5). Additionally, to determine the particular time points x TBI interaction, some results were evaluated using two-way ANOVA followed by Bonferroni’s multiple comparisons post hoc test (Figs. 1, 2, 3). All the statistical analyses mentioned above were performed with GraphPad Prism ver. 8.1.1 (330) (GraphPad Software, Inc., San Diego, USA).

## Results

### Time-dependent changes in *XCL1* mRNA expression in the cortex, thalamus and hippocampus of mice after TBI

We observed a strong and significant increase in *XCL1* mRNA expression in all the analyzed brain structures (cortex, thalamus and hippocampus; Fig. 1). The level of *XCL1* mRNA after TBI was significantly increased at every tested time point (24 h, 4 days, 7 days, 2 weeks and 5 weeks) compared to the level after the sham operation, except for the 24 h and 5 weeks in the thalamus and 24 h and 4 days in hippocampus. In the cortex, the highest upregulation was detected 2 weeks after trauma [12.99 ± 2.53 (*t*_8_ = 4.74; *p* = 0.0089)], after which the effect started to decrease through week 5 [4.26 ± 0.79 (t_10_ = 4.03; *p* = 0.0087)]. The two-way ANOVA confirmed a significant interaction between the considered time points and TBI procedure (*F*_4,55_ = 12.42; *p* < 0.0001) (Fig. 1a). In the thalamus (Fig. 1b), we also noticed an increase in *XCL1* mRNA expression—the highest value was observed at 7 days after injury [3.15 ± 0.7 (*t*_14_ = 2.94; *p* = 0.0181)]. The two-way ANOVA confirmed a significant interaction between the considered time points and TBI procedure (*F*_4,56_ = 3.88; *p* = 0.0075).The strongest upregulation of the chemokine*,* which was maintained at a high level from day 7 [18.6 ± 5.86 (*t*_14_ = 3.00; *p* = 0.0199)] until week 5 [18.05 ± 4.7 (*t*_9_ = 3.62; *p* = 0.0223)], was observed in the hippocampus. The two-way ANOVA confirmed a significant interaction between the considered time points and TBI procedure (*F*_4,55_ = 4.56; *p* = 0.0030) (Fig. 1c).

### Time-dependent changes in *XCR1* and *ITGA9* mRNA expression in the cortex, thalamus and hippocampus of mice after TBI

Analysis of mRNA levels of the XCL1 receptor *XCR1* similarly revealed its upregulation after TBI in all the tested brain structures (Fig. 2a–c). In the cortex, the level of *XCR1* was elevated at every tested time point (24 h, 4 days, 7 days, 2 weeks and 5 weeks). The cortical level of *XCR1* (Fig. 2a) started to increase 24 h after TBI and reached its highest value at day 4 [7.89 ± 0.97 (*t*_12_ = 7.05; *p* < 0.008)]; the expression of *XCR1* progressively decreased but remained significantly elevated at week 5. The two-way ANOVA confirmed a significant interaction between the considered time points and TBI procedure (*F*_4,50_ = 5.23; *p* = 0.0013) (Fig. 2a). The *XCR1* mRNA level in the thalamus started to grow 24 h after TBI [1.71 ± 0.25 (*t*_10_ = 2.57; *p* = 0.0380)] and reached its highest value 4 days after TBI [3.08 ± 0.43 (*t*_12_ = 4.53; *p* = 0.0038)] but remained significantly elevated until 7 days (Fig. 2b). After that time point, the level decreased. The two-way ANOVA confirmed a significant interaction between the considered time points and TBI procedure (*F*_4,53_ = 4.84; *p* = 0.0021) (Fig. 2b). The elevated level of *XCR1* mRNA in the hippocampus was observed 24 h and 7 days after injury [16.98 ± 4.36 (*t*_12_ = 3.66; *p* = 0.0145)], after which it began to decline but remained significantly elevated until 2 weeks after injury (Fig. 2c).

The mRNA expression of the second receptor that was studied (Fig. 2d–f), the *ITGA9*, was increased in two of the selected brain areas**.** In the cortex (Fig. 2d), the increase started 4 days after brain injury [1.91 ± 0.22 (*t*_14_ = 3.88; *p* = 0.0034)], and remained at a significant level until week 5. The two-way ANOVA confirmed a significant interaction between the considered time points and TBI procedure (*F*_4,58_ = 3.35; *p* = 0.0155) (Fig. 2d). The maximal expression of *ITGA9* in the hippocampus (Fig. 2f) was also observed at 4 days after trauma [2.98 ± 0.64 (*t*_12_ = 3.04; *p* = 0.0269)], and the level of *ITGA9* remained at a significant level until week 2. We did not notice any changes in the mRNA of this receptor in the thalamus (Fig. 2e).

### Time-dependent changes in the XCL1, XCR1 and ITGA9 protein levels in the cortex, thalamus and hippocampus of mice after TBI

Here, we analyzed the XCL1 protein using ELISA at two selected time points after TBI (24 h and 7 days) in the cortex, thalamus and hippocampus. The XCL1 level in the cortex (Fig. 3a) were significantly elevated at 24 h [3.15 ± 0.18 (*t*_9_ = 9.67; *p* < 0.0001)]. The two-way ANOVA confirmed a significant interaction between the considered time points and TBI procedure (*F*_1,19_ = 13.48; *p* = 0.0016) (Fig. 3a). In the thalamus, there was a significant increase in the chemokine levels only at the 24 h time point [1.22 ± 0.08 (*t*_10_ = 2.31; *p* = 0.0452)] (Fig. 3b). The hippocampal activation of XCL1 was significantly increased at both the selected time points, namely 24 h [7.2 ± 1.44 (*t*_10_ = 4.29; *p* = 0.0074)] and 7 days [3.61 ± 0.7 (*t*_10_ = 3.71; *p* = 0.0138)], after TBI. The two-way ANOVA confirmed a significant interaction between the considered time points and TBI procedure (*F*_1,20_ = 4.99; *p* = 0.0369) (Fig. 3c).

Moreover, we performed Western blot analysis of both the XCL1 receptors, namely, XCR1 and ITGA9, at two selected time points after TBI (24 h and 7 days) in the same brain structures. XCR1 showed no changes at the protein level in the cortex (Fig. 3d), thalamus (Fig. 3e), and hippocampus (Fig. 3f). Similar results were obtain for ITGA9 in the cortex (Fig. 3g), thalamus (Fig. 3h), and hippocampus (Fig. 3i).

### XCL1, XCR1 and ITGA9 mRNA and protein levels in primary glial cell cultures

In murine primary microglial and astroglial cell cultures, we detected the basal expression of XCL1 mRNA and protein (ELISA) (Fig. 4a, b). There are no significant changes in the protein (Fig. 4b) level of XCL1 24 h after LPS stimulation in the cultures of either cell type, however there is an upregulation of XCL1 in astroglial cells mRNA after LPS stimulation [3.90 ± 0.93 (*t*_6_ = 3.09; *p* = 0.0353)] (Fig. 4a).

We also detected the basal mRNA and protein levels (Western blot) of both receptors, XCR1 (Fig. 4c, d) and ITGA9 (Fig. 4e, f). In the primary microglial cell cultures, no changes were observed in the mRNA and protein level of XCR1 24 h after LPS stimulation (Fig. 4c). In the primary astroglial cell cultures, there were no changes in the mRNA of *XCR1*; however, the protein level of this receptor was significantly decreased 24 h after LPS stimulation [0.62 ± 0.05 (*t*_9_ = 5.37; *p* = 0.0005)] (Fig. 4d). In the primary microglial and astroglial cell cultures, there was an increase in the *ITGA9* mRNA levels after LPS treatment in both cell populations [microglia: 1.95 ± 0.33 (*t*_10_ = 2.77; *p* = 0.0276), astroglia: 1.45 ± 0.11 (*t*_10_ = 3.32; *p* = 0.0110)] (Fig. 4e), the protein level of the *ITGA9* increased 24 h after LPS stimulation in astroglial cells population [1.32 ± 0.12 (*t*_10_ = 2.61; *p* = 0.0440)] (Fig. 4f).

### The effect of XCL1 on the mRNA levels of pro- and anti-inflammatory cytokines in primary glial cell cultures

Primary microglial and astroglial cell cultures were stimulated with XCL1 (200 ng/ml). Our further analysis performed at 2 and 6 h after XCL1 administration proved the presence of mRNAs encoding pro- (*IL-1ß, IL-18, IL-6, CCL3, CCL4, CCL9)* and anti- (*IL-10, IL-18BP*) inflammatory cytokines in both primary microglia and astroglial cell cultures. The mRNA expression of pro-inflammatory interleukins was not affected by XCL1 administration in microglial as well as astroglial cell cultures. The expression of *CCL4 and CCL9* was unchanged by XCL1 administration in both cell cultures (Fig. 5i–l). The level of *CCL3* was slightly lowered 2 h after XCL1 administration in microglia [0.85 ± 0.04 (*t*_6_ = 3.25; *p* = 0.0174)] (Fig. 5g), but it might be not biologically relevant. However, there was no changes in expression of *CCL3* mRNA after XCL1 in astrocytes (Fig. 5h). The mRNA levels of the anti-inflammatory cytokines *IL-18BP* and *IL-10* were unchanged after treatment with the XCL1 in both cell cultures (Fig. 5m–p).

## Discussion

The results of the present study show, for the first time, that XCL1 is highly spatiotemporally increased at the mRNA and/or protein levels in the cortex, thalamus and hippocampus after TBI. Additionally, the XCL1 receptors, XCR1 and ITGA9, which are present in the all studied brain structures, are highly upregulated at the mRNA level; however, their protein levels do not exhibit significant changes. Our in vitro studies revealed that murine primary microglial and astroglial cells expressed XCL1 and both its receptors, however this chemokine is not upregulated after cellular activation. Our findings indicate that XCL1/XCR1 and XCL1/ITGA9 seem to be key signaling pairs that can participate in many aspects of secondary brain injury. Since the XCL1 can be one of the important triggers of secondary injury after TBI, therefore we proposed this chemokine as a good target for pharmacological intervention [31–34].

Initially, it was shown that in the periphery, XCL1 is produced by subsets of T and NK cells during inflammation and leads to chemotaxis of these cells by binding to XCR1 [12]. This is in line with our in vitro results showing that activated microglia and astroglia are not, as can be expected, the main source of XCL1 after brain injury. The strongly increased XCL1 level observed after nervous system injury may be due to the secretion of this chemokine by neurons, as already suggested [11]. It seems that XCL1 is an important player in many immune responses [35]. It has been already identified in patients with several inflammatory diseases, including Crohn’s disease [36], HIV-1 infection [37] and rheumatoid arthritis [38, 39]. Importantly, also in a cerebral tissue of patients with posttraumatic brain contusions [40]. Some findings highlight the possible significance of the XCL1/XCR1 pathway in maintaining gut homeostasis, which can define this axis as an innovative potential therapeutic target for the treatment of human intestinal immune disorders [41]. The upregulation of XCL1 was also described in mice, first by Koedel et al. in the cortex 72 h after cold-induced cortical injury [42]. Moreover, Zychowska et al. showed that XCL1 is spinally upregulated in a mouse model of diabetic neuropathy and that its neutralization results in a reduction of hypersensitivity [10]. Recently, Matsumoto et al. [15] showed that XCL1- and ITGA9-neutralizing antibodies abrogated disease progression in experimental autoimmune encephalomyelitis and suggested XCL1/ITGA9 axis as an important signaling pair for homeostatic functions. We demonstrated the spatiotemporal upregulation of XCL1 expression in all the tested brain areas (cortex, thalamus, hippocampus), which begins shortly after TBI and, in some structures, persists until up to 5 weeks after injury. For our research, we selected brain structures that were previously suggested to be especially vulnerable to TBI [43–45]. In 2020, Mohamed et al. studied a rat model of closed head diffuse injury by diffusion tensor imaging and confirmed that TBI leads to widespread and persistent microstructural changes within the white and gray matter of the brain [46]. Moreover, like the cortex, also hippocampus and thalamus appear to be susceptible to ongoing post TBI pathology. The authors also confirmed volumetric changes in these two areas. Additionally, they observed increased microglial activation in the cortex, thalamus, and hippocampus, even up to 30 days [46, 47]. These results are consistent with those obtained using positron emission tomography, which verified increased inflammation in the thalamus after TBI [48]. Since the cortex is the site of the impact, as expected, significant changes in XCL1 were observed in this region. However, we also provide the first evidence of the profound XCL1 response in the thalamus and hippocampus. Many studies have shown that within the first hours after TBI, increased neuronal excitability and reduced GABAergic inhibitory transmission are observed, which entail astroglia and microglia activation [49]. The microglia are approximately 5–20% of the total glial cell population [50, 51] and play an important immunological role in the CNS [51]. Astrocytes occupy 30% of the brain volume (region dependent) and constitute 30–65% of all the glial cells [50], which makes these cells, together with neurons, the largest population of cells in the brain [52, 53]. Published data provide evidence that the inflammation observed in TBI is associated with thalamocortical white matter damage and profound glial activation, which colocalize with axonal abnormalities [54]. Our results prove that XCL1 is strongly upregulated shortly (24 h) after TBI, therefore we hypothesize that neuronally produced XCL1 acts in an autocrine manner via neuronal XCR1 and ITGA9 to trigger neuronal activation, which in turn results in glial activation. This observation corresponds well with our previously published results showing that activation of microglia and astroglia starts on day 7 after TBI [8]. Although our results show that upon in vitro conditions XCL1 does not directly activate glial cells, there are evidences that in vivo the situation may differ. It was already shown that intrathecally administrated XCL1 induced microglia activation and proliferation [10], therefore there is still a need for more research focused on XCL1 role in TBI. Already is known that, the disruption of the homeostatic interactions of the CX3CL1 (produced mainly by neurons) and CX3CR1 (present mainly on microglia) axis in the context of neuron-microglia/astroglia communication is important during the pathogenesis of several diseases, including TBI [55–58]. In addition, CX3CL1, CCL2, CXCL8 in the CSF and/or plasma of TBI patients correlate with poorer outcome, therefore they have been proposed as biomarkers (reviewed in [59]). The level of XCL1 in the CSF and plasma in TBI patients remain to be study, however importantly in 2020 this chemokine was selected as biomarker for malignant transformation [60].

Recent data strongly support the idea that microglia play both beneficial and harmful roles [61, 62]. Microglia can prevent neuronal injury and restore tissue integrity by releasing anti-inflammatory/neurotrophic factors and removing cellular debris. On the other hand, the development of an uncontrolled and highly reactive microglial activation state after brain injury results in the release of pro-inflammatory factors that contribute to neuronal dysfunction and death [61, 62]. Similarly, reactive astrocytes are capable of producing pro‐inflammatory factors and can degrade the extracellular matrix and cause further disrupt the BBB [53]; however, they are also capable of producing factors that promote regeneration [63]. Our results provide evidence that XCL1/XCR1 and XCL1/ITGA9 axes can participate in immune response after TBI. XCR1 was identified in 1995 as orphan receptor GPR5 [64] and, for the long time, was thought to be the only receptor for XCL1 in mice [65, 66]. It has been suggested that XCR1 is expressed in murine dendritic cells [67], T cells, B cells and neutrophils [68] but not in macrophages [69]. However, subsequent studies have proven that XCR1 is present on mononuclear cells [38, 70]. Our results obtained from primary cell cultures provide evidence that XCR1 is present on microglial and astroglial cells. After TBI, the mRNA level of *XCR1* is highly upregulated in all the studied brain structures; however, its protein level changes remain on undetectable level. Therefore, we can assume that the exposure of these receptors to XCL1 leads to a rapid decrease in the number of cell-surface binding sites. What is also important, XCL1 can also act through ITGA9 [15], so by one of the less studied integrins that facilitates accelerated cell migration [71]. It was already shown that blocking ITGA9 has beneficial effects in mouse models of arthritis [71] and experimental autoimmune encephalomyelitis [21]. Our studies indicate that in primary microglial and astroglial cell cultures, XCL1 does not induce production of pro-inflammatory cytokines such as CCL3, CCL4, CCL9, IL-1β, IL-18, IL-6 directly. Additionally, XCL1 administration does not influence the mRNA levels of an anti-inflammatory IL-1RA, IL-10 in both microglia and astrocytes. These findings are surprising but also extremely important as they highlight that XCL1 may acts through neuronally localized receptors. Considering that XCR1 in the CNS is located mainly on neurons [10] and similarly ITGA9 is also present in neurons [20] it seems to be even more likely. Current literature suggests that modulating chemokine signaling, especially CCL2/CCR2, CCL5/CCR5 CXCL8/CXCR2, CXCL10/CXCR3, CXCL12/CXCR4, and CX3CL1/CX3CR1, may be beneficial in TBI treatment [59]. Our results, for the first time, draw attention to the significant role of the XCL1/ITGA9 axis in the cortex, hippocampus and thalamus after brain injury. Interestingly, according to the literature data, we can hypothesize that XCL1 signaling via ITAG9 might be neuro-protective [19] while signaling via XCR1 neuro-toxic [10]. However, there is still a lack of study to prove this hypothesis. If this is true, this data would mean that downregulating XCL1-XCR1 signaling while simultaneously up-regulating XCL1-ITAG9 signaling is a very tempting therapeutic strategy. Still requires clarification which intracellular pathways are involved through signaling via XCL1-XCR1 and which through signaling via XCL1-ITAG9 in the CNS. So far, it was shown that XCL1-XCR1 evoked the induction of c-Fos, pERK and p38MAPK in brainstem [11]. In case of XCL1-ITGA9 axis there was shown, that ITGA9 was acting through FAK/Src-Rac1/RhoA signaling in human liver cell line [72]. It was also proved that that ITGA9 depletion suppresses breast cancer tumor growth and metastasis by promoting β-catenin degradation through the ILK/PKA/GSK3 pathway [73]. The modulating of XCL1 activated pathways has the potential to result in therapeutic benefit not only in TBI, but also in other neuroinflammation-related diseases, however, this hypothesis requires additional, in-depth investigation.

## Conclusion

Treatments for brain injury are a major medical need, so new approaches based on innovative potential therapeutic targets are urgently needed. The results of our research provide the first evidence that in the early phases of TBI (24 h), XCL1 is highly upregulated not only in a cortex, but also in thalamus and hippocampus. Therefore, this chemokine can be one of the immune triggers of secondary injury after TBI, therefore should be considered as an important chemokine that may play a pivotal function during brain injury. Based on the available literature and our results, we suggest that XCL1 deserves further study, especially because XCR1 and ITGA9 seem to be important novel targets with beneficial properties for pharmacological intervention after brain injury.
